# Structural variability and differentiation of niches in the rhizosphere and endosphere bacterial microbiome of moso bamboo (*Phyllostachys edulis*)

**DOI:** 10.1038/s41598-021-80971-9

**Published:** 2021-01-15

**Authors:** Zong-Sheng Yuan, Fang Liu, Zhen-Yu Liu, Qiu-Liang Huang, Guo-Fang Zhang, Hui Pan

**Affiliations:** 1 Institute of Oceanography, Minjiang University, Minhou County, Fuzhou, 350108 Fujian China; 2 College of Life Sciences, Fujian Agriculture and Forestry University, Fuzhou, 350002 Fujian China; 3 College of Plant Protection, Shandong Agricultural University, Tai’an, 271018 Shandong China; 4 College of Forestry, Fujian Agriculture and Forestry University, Fuzhou, 350002 Fujian China

**Keywords:** Ecology, Microbiology, Ecology

## Abstract

The plant microbiota play a key role in plant productivity, nutrient uptake, resistance to stress and flowering. The flowering of moso bamboo has been a focus of study. The mechanism of flowering is related to nutrient uptake, temperature, hormone balance and regulation of key genes. However, the connection between microbiota of moso bamboo and its flowering is unknown. In this study, samples of rhizosphere soil, rhizomes, roots and leaves of flowering and nonflowering plants were collected, and 16S rRNA amplicon Illumina sequencing was utilized to separate the bacterial communities associated with different flowering stages of moso bamboo. We identified 5442 OTUs, and the number of rhizosphere soil OTUs was much higher than those of other samples. Principal component analysis (PCA) and hierarchical clustering (Bray Curtis dis) analysis revealed that the bacterial microorganisms related to rhizosphere soil and endophytic tissues of moso bamboo differed significantly from those in bulk soil and rhizobacterial and endosphere microbiomes. In addition, the PCA analyses of root and rhizosphere soil revealed different structures of microbial communities between bamboo that is flowering and not flowering. Through the analysis of core microorganisms, it was found that *Flavobacterium*, *Bacillus* and *Stenotrophomonas* played an important role in the absorption of N elements, which may affect the flowering time of moso bamboo. Our results delineate the complex host-microbe interactions of this plant. We also discuss the potential influence of bacterial microbiome in flowering, which can provide a basis for the development and utilization of moso bamboo.

## Introduction

There are various microorganisms in the plant growth ecosystem that are attached to the surface or inside of plant. These microbial groups are collectively referred to as the plant microbiome, including bacteria, fungi, archaea, protozoa and viruses^1–4^. Studies have shown that the interaction between microorganisms and plants plays an important role in plant growth and development^3,5,6^, stress resistance^7,8^, nutrient uptake^9^ and sustainable production^10,11^. In particular, the bacterial microbiome can improve the ability of plant to transport nutrients, improve their utilization, increase tolerance of plants to adversity, and promote their resistance to stress, thereby affecting plant growth and yield^12,13^. In addition, bacterial microorganisms can affect plant growth by affecting the effectiveness of plants to take up nitrogen and regulate their flowering time by secreting tryptophan^14^. These effects show that the interaction between soil microbiome, soil exudates and plant physiology can dynamically affect rhizosphere community and change flowering phenotype of plants through a complex feedback mechanism^14^.

Moso bamboo, which belongs to the family Poaceae, is an important economic species, primarily distributed in Asia–Pacific, America and Africa^15^. The area of bamboo forests in China is approximately 4,430,100 ha, and they have a very important position in China's forest resources and production^16^. Moso bamboo is a perennial plant with an extremely fast growth rate, and it will only flower during 10 or even 100 years of growth^17,18^. Unlike other species, the flowering of moso bamboo is uncontrollable and unpredictable. The process is often accompanied by large-scale flowering in which the plants set seeds and die, which can result in the bamboo forest degradation leading to the economic and ecological losses.

There are several hypotheses on the frequency of flowering in bamboo, such as the period, environmental, comprehensive, stimulus, and free radical theories^18–22^. However, the physiological and genetic mechanisms of bamboo flowering remain unclear. Current studies have shown that the microbiome of moso bamboo changes with space and time and affects availability of soil nitrogen, thereby affecting the growth and reproduction of plant^14^. Therefore, the changes in microbiome may affect flowering of moso bamboo. In this study, we evaluate the differentiation of niches of microbiome of bacterial communities associated with the rhizome, roots, leaves, and rhizosphere and bulk soil of flowering and nonflowering moso bamboo using 16S rRNA Illumina sequencing. We evaluated the impact of microhabitat on rhizosphere and endosphere microbiomes and predicted the functions of rhizosphere and endosphere microbiomes based on their classification. We identified specific bacterial groups related to the flowering of moso bamboo. Our study provides a basis to cultivate forests of moso bamboo through the modification of its rhizosphere and endosphere microbiomes.

## Results

### Alpha rarefaction curves and alpha diversity

**Figure 1:**
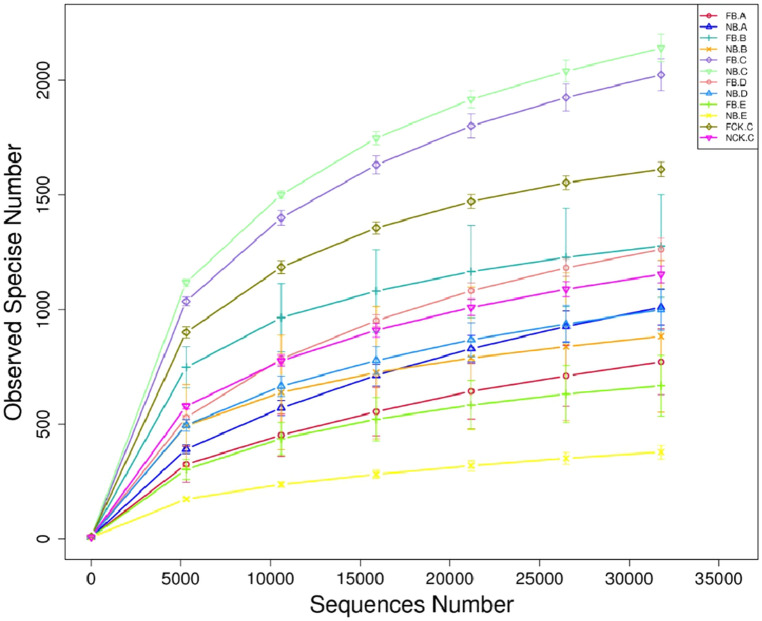
Average Good’s coverage estimates (%) of each moso bamboo plant compartment. Rarefaction curves were assembled showing the number of OTUs, defined at the 97% sequence similarity cut-off in mothur, relative to the number of total sequences.

**Figure 2:**
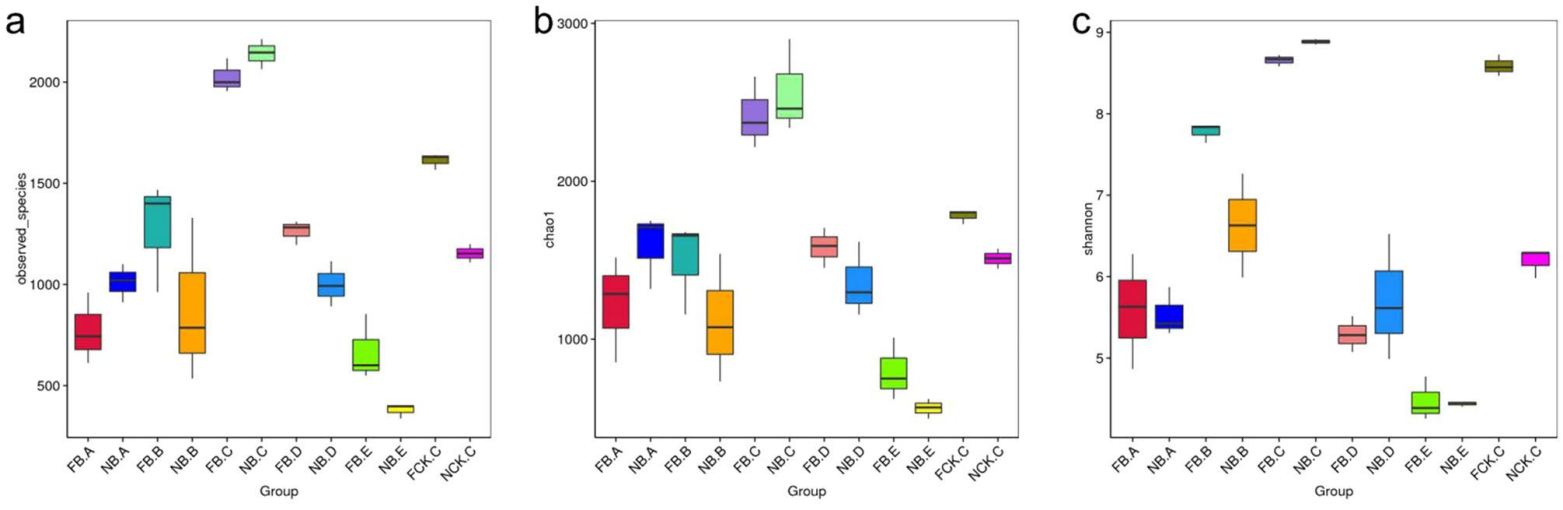
Alpha diversity estimates of the bacterial communities. (**a**) Number of observed OTUs). (**b**) Chao1 indices. (**c**) Shannon diversity indices. Alpha diversity estimates represent 3 biological replicates for the rhizosphere soil and root, rhizome, leaf samples were calculated in mothur with 10,000 iterations.

Based on the high-throughput sequencing of bacterial 16S rRNA gene V5-V7, we analyzed the rhizosphere and endosphere bacterial microbiome of flowering and nonflowering moso bamboo. Therefore, for DNA extraction flowering rhizome (FB.A), root (FB.B), rhizosphere soil (FB.C), new leaf blades (FB.D) and old leaf blades (FB.E),and rhizome (NB.A), root (NB.B), rhizosphere soil (NB.C), new leaves (NB.D), old leaves (NB.E), bulk soil (NCK.C) from nonflowering were sampled. A total of 3,076,450 pairs of double-ended sequences (Reads) were obtained by sequencing, and 3,013,256 high quality sequences (Clean tags) were obtained after stitching and filtering the reads. The number of clean tags in each sample was 57,977–98,163, and the sequences were clustered after quality control based on 97% sequence similarity to obtain 5442 bacterial OTUs. By constructing a dilution curve, the results showed that the diversity of rhizobacteria was much higher than that of endogenous bacterial communities. Most root endogenous samples were saturated at approximately 750–1300, and rhizosphere samples were saturated at only approximately 2000 OTUs (Fig. 1). A statistical difference analysis of alpha diversity also inferred a similar degree of OTU richness (Fig. 2a). In addition, the Chao 1 and Shannon index indicate a significant difference between the two groups FB.A-FCK.C and FB.E-NB.B (Fig. 2b,c), indicating that the sequencing depth is sufficient to reliably describe bacterial microbiome associated with these bamboo and soil samples.

**Figure 3:**
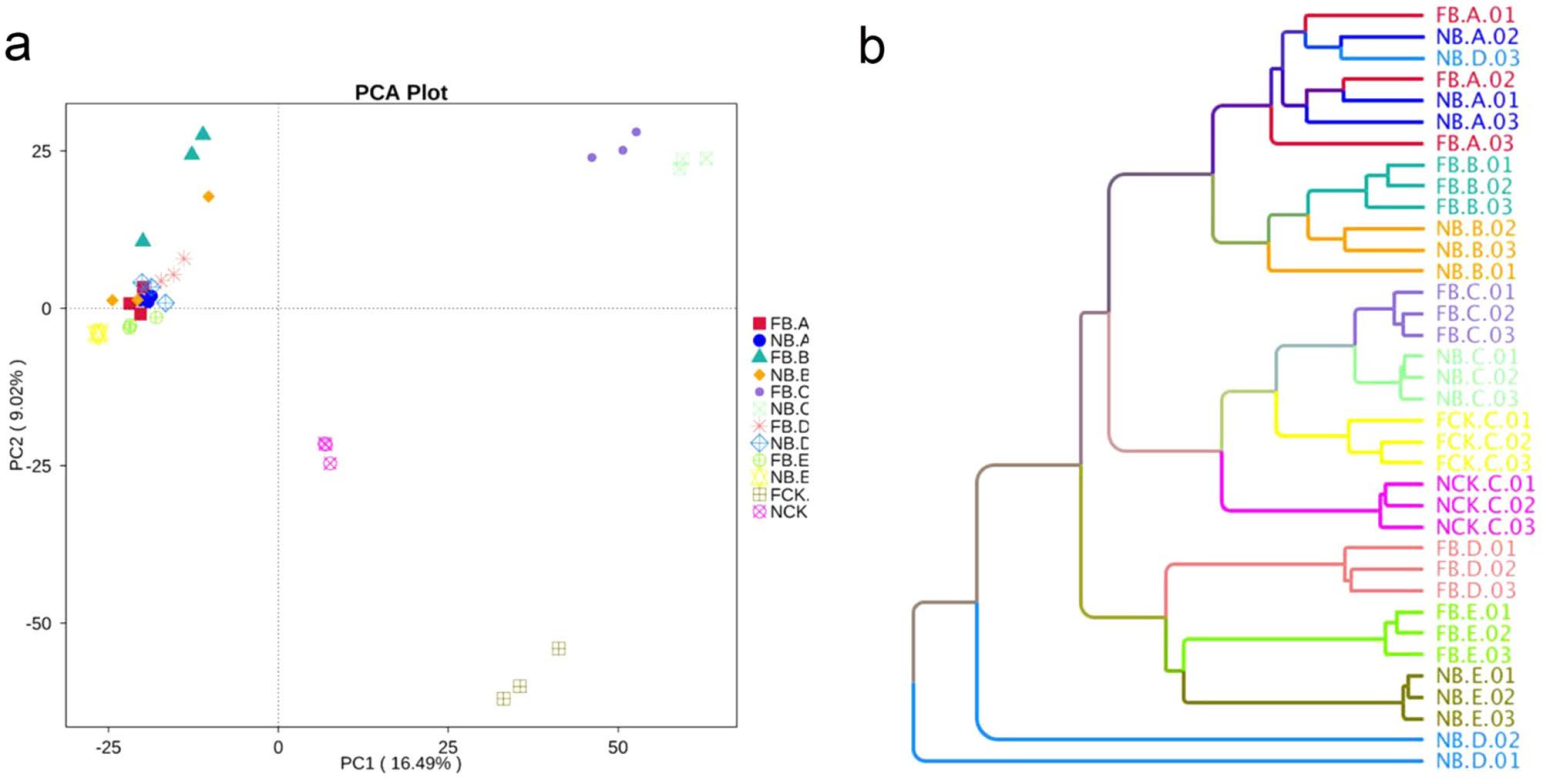
Plant compartment drives the composition of the bacterial communities at the OTU level. (**a**) Principle component analysis (PCA) of square-root transformed samples based on rarefaction to 2000 reads per sample. (**b**) Hierarchical clustering (group average linkage) of the samples based on Weighted Unifrac. PCA and hierarchical clusters were based on 3 biological replicates (rhizosphere soil and root, rhizome, leaf samples) and were constructed in PRIMER 7 with 10,000 iterations.

To compare the effects of different flowering states and microhabitats on microbial community structure of moso bamboo, the principal components PC1 and PC2 indicate an overlap of 16.49% and 9.02%, respectively (Fig. 3a). The three biological repeats of each flowering stage and tissue sample are closely clustered, indicating that our 16 sequencing results highly repeatable and reliable. PC1 is primarily classified from the stage of different tissue samples, and PC2 is classified from the flowering stage. In addition, hierarchical clustering at the OTU and genus levels showed that the rhizosphere soil, root and old leaf samples were completely clustered, but the bamboo rhizome sample was significantly different from the rhizosphere soil and old leaf samples (Fig. 3b).

### Core bacteria microbiome within each plant compartment

**Figure 4:**
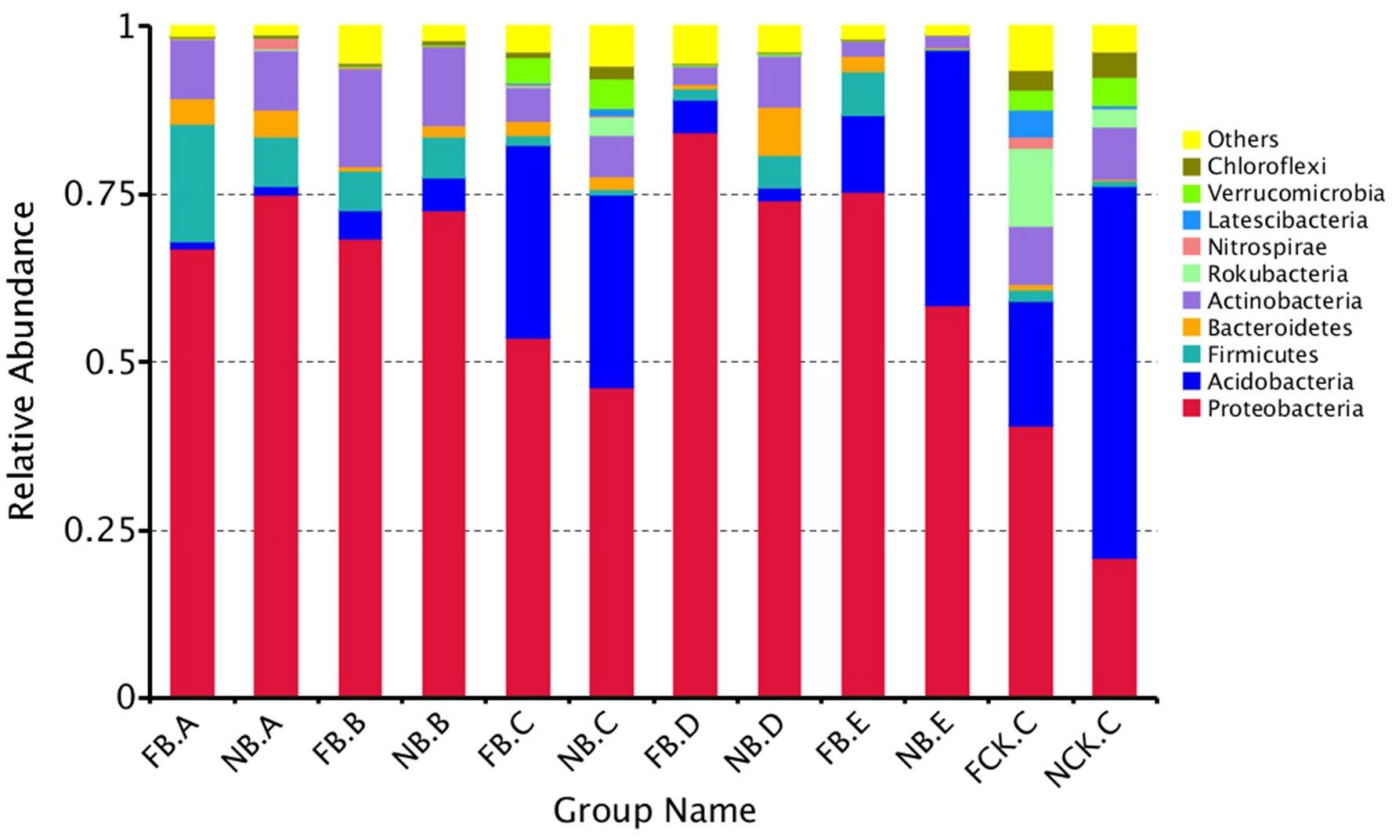
Dominant bacterial phyla detected in moso bamboo rhizosphere soil, root, rhizome, leaf compartments and bulk soils.

To further study the changes of specific taxa in the rhizosphere and endosphere bacterial microbiome of moso bamboo flowering and nonflowering stages, we compared the relative abundance of rhizosphere and endosphere bacterial microbiome at phylum level (Fig. 4). Among them, Proteobacteria, Acidobacteria and Firmicutes are the primary ones that dominate the phylum level. As shown in Fig. 4, by comparing the ratio of abundance of rhizosphere bacterial communities, the relative abundance of Proteobacteria in the rhizosphere soil of flowering moso bamboo is relatively higher than that of nonflowering (P < 0.05), while those of Rokubacteria, Latescibacteria and Chloroflexi were reduced (P < 0.01). The richness of endophytic bacteria in the roots did not change significantly whether plants were flowering or not. In old leaves, Proteobacteria, Firmicutes, and Bacteroidetes increased in flowering moso bamboo, while Acidobacteria decreased (P < 0.05). In addition, in the new leaves, the abundance of Bacteroidetes decreased in flowering plants (P < 0.05). These results indicate that the richness of these specific bacterial communities at different flowering stages of moso bamboo vary.

**Figure 5:**
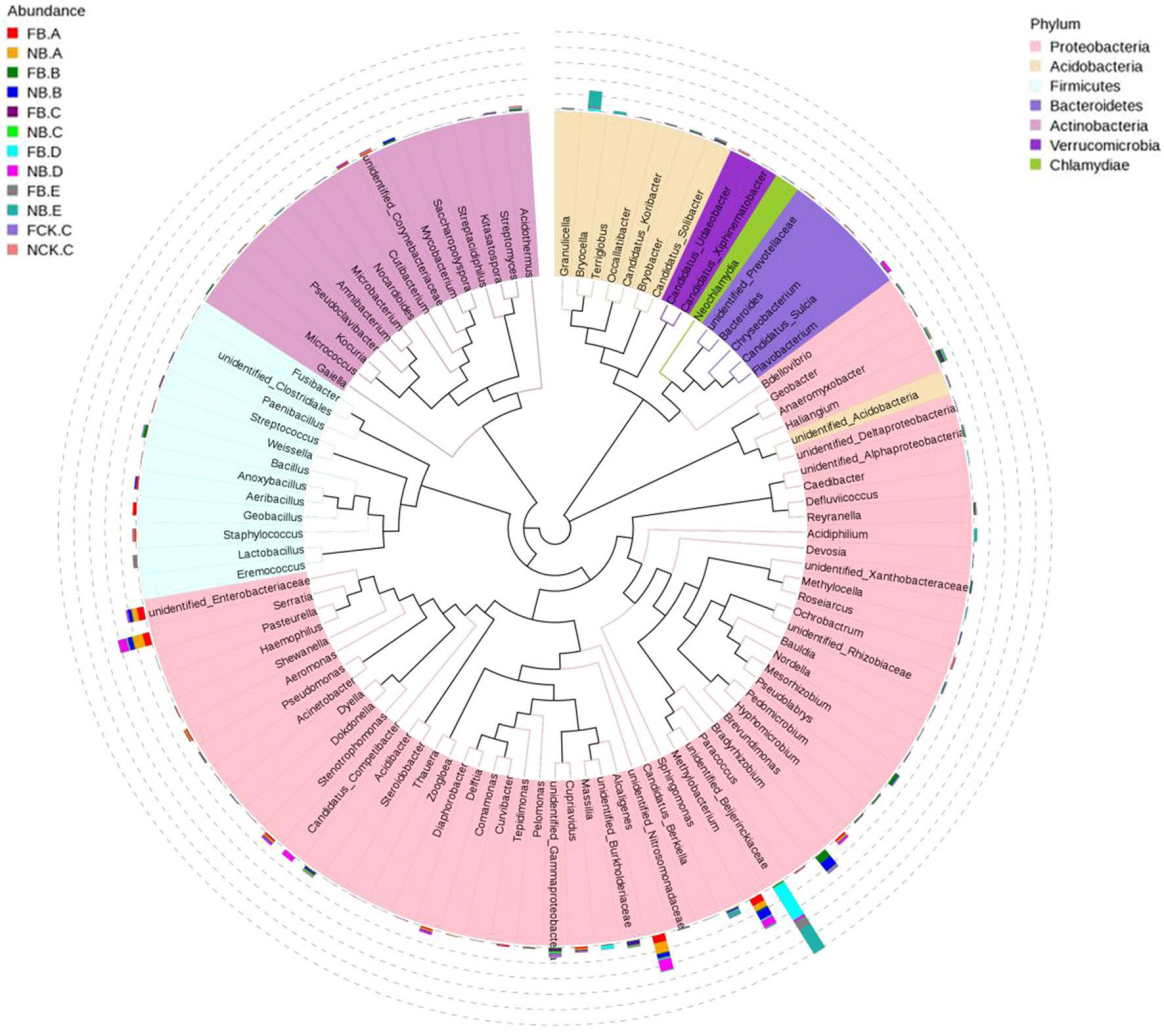
Top OTU members of the bacterial microbiome associated with the plant niches. Taxonomic dendrogram showing the core bacterial microbiome of each plant compartment. Color ranges identify genera within the tree.

To further understand the changes of these specific bacterial communities, we studied the phylogenetic relationships of species at the genus level through multiple sequence alignments to obtain representative sequences of the top 30 genera. As shown in Fig. 5, in the rhizosphere bacterial community of moso bamboo, *Geobacillus*, *Bacillus*, *Cupriavidus*, and *Haliangium* increased in richness after flowering. The abundances of *Geobacillus*, *Aeribacillus* and *Bacillus* increased in rhizome after flowering, but the abundance of *Comamonas* decreased (Fig. 5). In the root, the abundances of *Alcaligenes*, *Serratia*, Enterobacteriaceae, and *Aeribacillus* decreased in flowering moso bamboo, while the abundances of *Pseudolabrys* and *Bacillus* increased (Fig. 5). In the new leaves, the abundances of Beijerinckiaceae and *Massilia* significantly increased in flowering moso bamboo, while the abundances of *Alcaligenes*, *Competibacter*, *Methylobacterium* and *Serratia* decreased. Beijerinckiaceae, *Bryocella*, *Sphingomonas*, *Scidiphilium* and *Terriglobus* significantly decreased in old leaves from flowering plants (Fig. 5). These abundances changed in flowering and nonflowering moso bamboo patterns, indicating that the rhizosphere soil and plant endogenous bacterial community have some relationship with the flowering of moso bamboo.

### PICRUSt analysis

**Figure 6:**
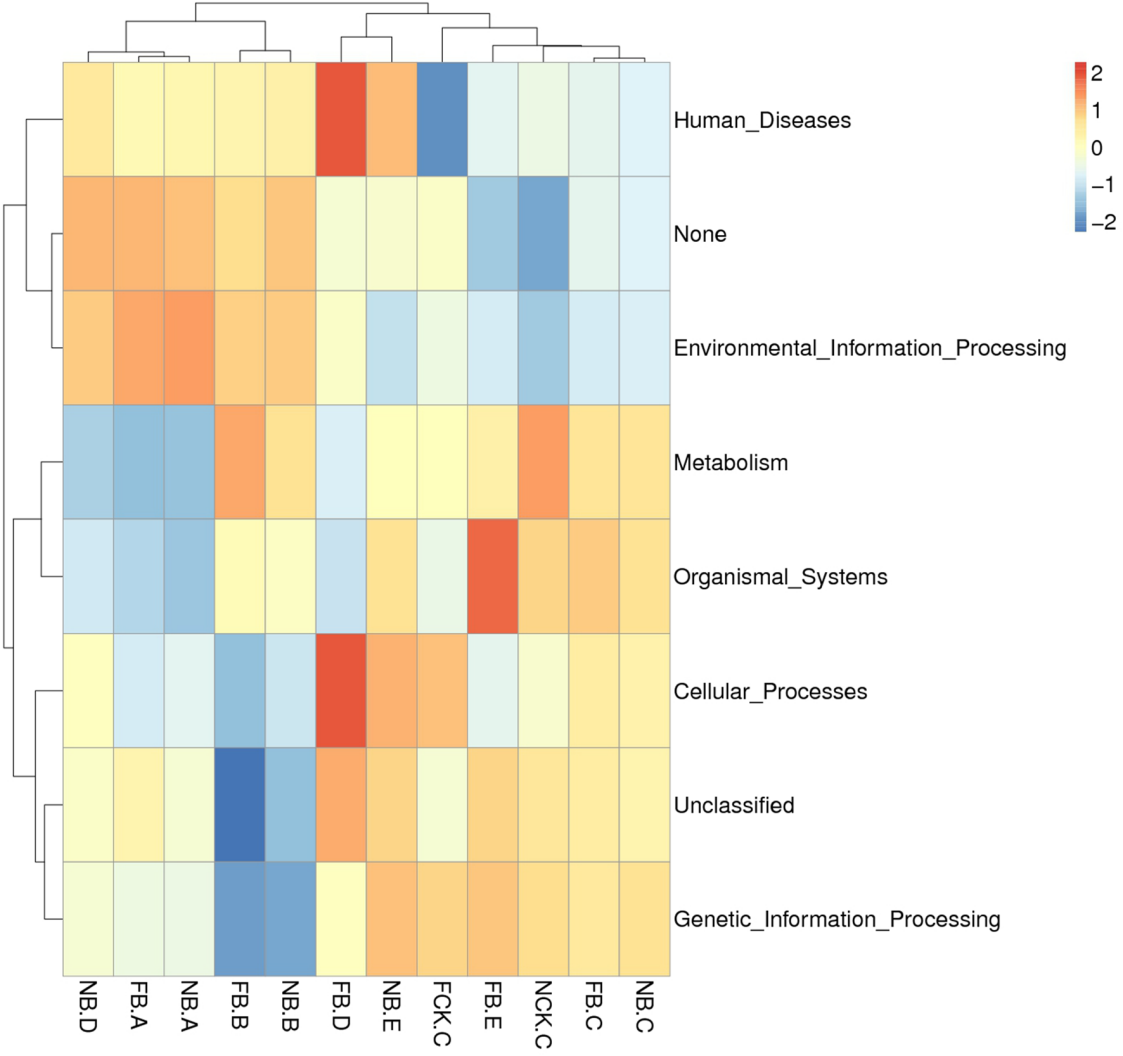
PICRUSt analysis of the bacterial microbiome in each plant compartment. The top 35 the bacterial microbiome and their abundance information in each sample were mapped and clustered from the functional difference level.

To further understand the function of specific bacterial communities, a PICRUSt analysis was performed, and we identified the heat maps based on the top 35 selected abundance functions, their abundance information in each sample and clusters from the functional difference level. There are obvious differences in the metabolism, biological processes of the cells, tissue system of different tissues and rhizosphere microorganisms in the moso bamboo samples at different flowering stages (Fig. 6), indicating that there might be some singular bacteria that affect the growth and development of bamboo, thus affecting the flowering of moso bamboo.

## Discussion

### Rarefaction curve, alpha diversity and PCoA analysis

By comparing the different OTU sparse curves of moso bamboo rhizosphere soil and endophytic tissue, we found that the rhizosphere soil showed a uniform sparse curve, and the change in sparse curve was much higher than that of sample of endophytic tissue, particularly compared with that of the leaf tissue (Fig. 1). These results show that the rhizosphere bacterial community of moso bamboo is highly abundant, and they were consistent with those of poplar^23^, tomato^24,25^ and other species, indicating that the rhizosphere bacterial microbiome was aggregated. In addition, the sparse curve depicted by the OTU of the rhizome tissue has a highly uneven curve (Fig. 1), which may be caused by the uneven colonization of the endosphere bacterial microbiome of the moso bamboo root, which may be due to root secretions and mucus-derived nutrients that attract numerous organisms to the rhizosphere environment; however, plant-related bacteria must be highly competitive to successfully colonize the root zone^26–29^. There are abilities to take up different nutrients and secrete derivatives between root systems of moso bamboo, as well as the interaction with roots and endosphere bacterial microbiome, which leads to differences in the endosphere bacterial microbiome and rhizome of moso bamboo^30,31^.

In addition, the endosphere bacterial microbiome structure has more changes compared with the rhizosphere bacterial microbiome (Fig. 3). This result may be owing to the use of two different kits for rhizosphere soil samples and plant tissues samples, resulting in more changes in the endophytic bacterial microbiome^32^. In addition, to compare the bacterial microbiome structure that exists in the plant compartment, the principal component analysis (PCA) and hierarchical clustering (Bray Curtis dis) shown in Figs. 1 and 3, the bacterial microbiome of rhizosphere soil and rhizome show significant difference between flowering and nonflowering stages. An ANOSIM analysis also shows this difference. Root deposits and exudates of the host plants in root zone promote chemical attraction and colonization of the rhizosphere soil and root plane, thereby forming a unique, highly rich and diverse rhizosphere microbiome^33^, while the root system is an important tissue for growth, development and reproduction of moso bamboo, as well as nutrient and moisture uptake^34–36^. Our results have shown that there is a difference in the bacterial microbiome between the rhizosphere and rhizome of flowering and nonflowering moso bamboo. This may be related to whether the bamboo is in a flowering state.

### Drivers of microbiome niche differentiation

An additional analysis of rhizosphere and endosphere bacterial microbiome at the phylum level in moso bamboo indicated that the main dominant bacteria of rhizosphere are Proteobacteria, Acidobacteria and Firmicutes (Fig. 4), This result is consistent with those of Arabidopsis^37^, rice^38^, poplar^23^ and soybean^39^ and indicates that the establishment of moso bamboo rhizosphere bacterial communities also follow the general rules of microbial community establishment. Among them, Proteobacteria and Acidobacteria are the main components of rhizobacteria. The ratio of Proteobacteria and Acidobacteria in the rhizobacteria has been proven to be an indicator of soil nutrient content. Proteobacteria is associated with nutrient-rich soil, while Acidobacteria is associated with nutrient-poor soils^40–42^. This is similar to the main composition of rhizosphere soils, such as those of Arabidopsis^36^, rice^38^, and poplar^23^. The relative abundance of rhizosphere soil is higher than that of bulk soil (FCK.C and NCK.C), indicating that there are enriched bacterial communities around the roots of moso bamboo. These bacterial communities may be specific to moso bamboo, or they may be a ubiquitous bacterial community. It has been reported that microorganisms enriched in soil roots play a very important role in plant production and development, including plant stress resistance, nutrient absorption and periodicity of response to plants^8^. At the phylum level, Proteobacteria and Acidobacteria are similarly enriched in the flowering and nonflowering moso bamboo rhizosphere. The proportion of Firmicutes in flowering moso bamboo in the rhizosphere is slightly greater than in nonflowering, while those of Rokubacteria, Latescibacteria and Chloroflexi are significantly reduced. Chloroflexi can utilize light energy to convert organic matter into energy at high temperature^43^, which may indicate that these significantly fewer bacterial communities are caused by the reaction of moso bamboo against biotic stress, abiotic stress, different nutritional conditions and plant immune responses. Therefore, it has potential interactions with different flowering phenotypes of moso bamboo. Additional research on these rhizosphere-specific bacteria will help to further understand the functionality of bacteria related to moso bamboo flowering.

In addition, at the phylum level, the richness of Rokubacteria and Nitrospirae was significantly reduced in rhizome after flowering, and the richness of Bacteroidetes and Verrucomicrobia was also significantly reduced in rhizome after flowering. The root systems are important tissues for the reproduction and nutrient absorption of moso bamboo. Endogenous bacteria colonize the plant tissues and depend on organic substances and other derivatives provided by plants to survive. The metabolites produced by their activities also play an important role in plant growth. Previous studies have shown that Nitrospirae is Gram-negative. Nitrifier as a type of nitrifying bacteria can oxidize nitrite to nitrate^44^. The diversity of function of Bacteroidetes depends on the genus. Bacteroidetes is increasingly regarded as a major member of the bacterial group that degrades a series of high molecular weight organic compounds, such as proteins and carbohydrates^45^.

Some members of Verrucomicrobia have recently been found to be capable of oxidizing methane and using it as the sole source of carbon and energy, which is of substantial significance to the carbon transfer of plants^46^. The bacteriophyta was a significant change in the richness of flowering bamboo and nonflowering bamboo, which could be involved in the uptake of nutrition and energy, particularly the nutrient elements in the soil.

The leaf is one of vegetative organs of plant. Its function is to synthesize organic matter through photosynthesis; the leaves provide the energy source for root system to absorb water and mineral nutrients from the outside through transpiration^47,48^. At the phylum level, the abundance of Bacteroidetes and Nitrospirae in new leaves of flowering moso bamboo decreased significantly compared with those that were not flowering, which also indicated that there was an energy imbalance in the leaves of flowering and nonflowering moso bamboo. This is consistent with a previous study on the photosynthetic efficiency of flowering moso bamboo leaves and the significant reduction of nutrient element accumulation, indicating that there are possible interrelated mechanisms.

Studies have shown that *Flavobacterium*, a member of the Bacteroidetes, represents an important part of the root and leaf-associated microbial community in plants. It is primarily capable of decomposing organic matter, and some species also catalyze nitrogen removal reactions^45^. After flowering, the abundance of *Flavobacterium* was significantly reduced in roots compared with those of nonflowering plants, which indicates that there was no abundant organic matter in the root of flowering bamboo, which resulted in a decrease in the abundance of *Flavobacterium*. There are many hypotheses about bamboo flowering. Among them, the nutrition theory has always been a topic of concern. Previous research found that the contents of organic matter and mineral elements in moso bamboo decreased after flowering, which is consistent with our results from the perspective of microbes, indicating that *Flavobacterium* may have an important effect on the flowering of moso bamboo.

*Bacillus* is a bacterial genus that has been described as containing plant growth-promoting rhizobacteria (PGPR) that shifted in response to the N content^49,50^. In our results, the abundance of Bacillus in the rhizosphere soil and plant tissues of flowering samples is significantly higher than that of nonflowering samples (more than sevenfold), indicating that flowering moso bamboo may obtain a more available nitrogen source. In addition, *Stenotrophomonas* is a denitrifying bacterium^14,50^, which is approximately four times more abundant in the roots of nonflowering moso bamboo than those that were flowering. The results of this study showed that nonflowering bamboo could convert nitrogen into an unusable nitrogen source. It has been reported that the availability of nitrogen in plants is closely related to the time of flowering, and the uptake of nutrients by plants involves complex biological processes. Changes in the abundance of bacteria, such as *Bacillus* and *Stenotrophomonas*, will change the effective utilization of nitrogen by plants^49,50^. Our research also found differences between these two important bacteria in the rhizosphere soil and tissues of flowering and nonflowering moso bamboo, indicating that the flowering of moso bamboo is closely associated with bacterial microbiome.

Our PICRUSt analysis also shows that the analysis of the bacterial function of moso bamboo in different samples of flowering and nonflowering involves plant metabolism, tissue system and cellular biological processes, which further verifies that the difference in endophytic bacteria in rhizosphere and plant tissue is related to the growth and development of moso bamboo, indicating that the changes in flowering and nonflowering stages are closely related to bacterial microbiome^8,34^. To verify the function of these specific bacteria, we will continue to closely analyze the functional verification of these microorganisms, which will aid in the functional study of effect of bacteria on flowering of moso bamboo.

## Materials and methods

### Plant materials and sampling

Moso bamboo tissues and rhizosphere soil were collected from Guilin City (117° 58′ 45″ E ~ 118° 57′ 11″ E; 26° 38′ 54″ N ~ 27° 20′ 26″ N), Guangxi Province, China in June 2019 to use as samples to extract DNA. At the time of sampling, the samples collected included rhizosphere soil, roots, rhizome and leaves. The moso bamboo trees were approximately 3.5–4.5 m tall on average. Three individual trees were selected.

### Processing of samples

The samples were processed as described by Beckers et al.^23^. Briefly, the root samples were removed from soil particles by shaking on a platform for 20 min at 120 rpm. The soil particles directly dislodged from roots represented the “rhizosphere soil” section. Subsequent “root” and “leaf” compartments were cleared of epiphytic bacteria by sequential washing (surface sterilization) with (a) sterile Millipore water (30 s), (b) 70% (v/v) ethanol (2 min), (c) sodium hypochlorite solution (2.5% active Cl– with 0.1% Tween 80) (5 min), and (d) 70% (v/v) ethanol (30 s) and finalized by rinsing the samples five times with sterile Millipore water. Finally, quadruple aliquots of each sample (1.5 ml) of the homogenized plant material (root or leaf) were stored for all moso bamboo individuals at − 80 °C until DNA was extracted.

### DNA extraction and sequencing

The total DNA was extracted from soil using a TIANGEN TIANamp Soil DNA Kit (DP336) according to the manufacturer’s instructions. DNA was isolated from roots and leaves using a Plant Genomic NDA Kit (DP305) according to the manufacturer’s instructions. The specific primer (799F [5′-AACMGGATTAGATACCCKG-3′] and 1391R [5′-GACGGGCGGTGWGTRCA-3′]) were used to amplify the V5-V7 hypervariable regions of the 16S rRNA gene. The PCR amplification of the V5-V7 regions from all compartments was amplified using polymerase chain reaction (S1000 Thermal Cycler, Bio-Rad, Hercules, CA, USA). Each reaction contained 10 ng of DNA, 2.5 μl 10 × PCR reaction buffer, 1.8 mM MgCl2, 0.5 μl dNTP mix, 0.5 μl of each primer (20 μmol), and 0.5 μl Taq polymerase. Cycling conditions included initial denaturation at 94 °C for 4 min, followed by 35 cycles of denaturation at 94 °C for 30 s, annealing at 52 °C for 45 s, and extension at 72 °C during 1.5 min; a final extension phase was performed at 72 °C during 8 min. Purified amplicons were sequenced on an Illumina NovaSeq instrument (Illumina Inc., San Diego, CA, USA) by Nanjing Novogene Bio Technology Co., Ltd. (Nanjing, China) utilizing the standard protocols.

### Statistical analysis

All samples were analyzed in triplicate. The data are presented as the mean ± standard error of the mean (SEM). Rarefaction curves that show the number of OTUs defined at the 97% sequence similarity cut-off relative to the number of total sequences were assembled in Mothur. An ANOVA was used to test the effect of the plant compartment (rhizosphere soil, roots, Rhizome and leaves) on the abundances of reads. Hierarchical clustering (based on Bray–Curtis dissimilarities) and principal component analyses (PCA) were performed in and displayed with PRIMER 7.
